# Epidemic Diphtheria: A Research on the Origin and Spread of the Disease from an International Standpoint

**Published:** 1899-03

**Authors:** 


**Epidemic Diphtheria: a Research on the Origin and Spread of the Disease from an International Standpoint.** By Arthur Newsholme, M.D. Pp. iv., 196. Swan Sonnenschein & Co., Limd. 1898.

This painstaking and valuable contribution to the epidemiology of diphtheria deserves careful study. The first part of the book is taken up with graphic charts showing the behaviour of diphtheria over a long series of years in towns and districts in all parts of the world. The second half of the book contains

Dr. Newsholme's review of the prevalence of diphtheria as shown in these charts, a discussion of the conditions determining pandemics of diphtheria, the relationship of rainfall and of soil to diphtheria, and a review of the position of preventive medicine in relation to the still-increasing prevalence of this disease. While Dr. Newsholme's conclusions place "waves of diphtheria-prevalence" on a plane above our control, the admission that diphtheria is spread chiefly by personal infection, which is, however, immensely more potent in epidemic than in inter-epidemic years, encourages the hope that present-day methods of control, including bacteriological supervision of throat-cases, should prove effectual in checking its spread.

